# Supplementary figures and images for: Complement Factor H and its C. elegans homolog regulate IFT52/OSM-6 and CNG channel localization in sensory neurons
Source: bioRxiv. 2025 Oct 13:2025.10.10.681644. Preprint. [Version 1] doi: 10.1101/2025.10.10.681644 (PMC12632885; doi:10.1101/2025.10.10.681644)

Figure S1

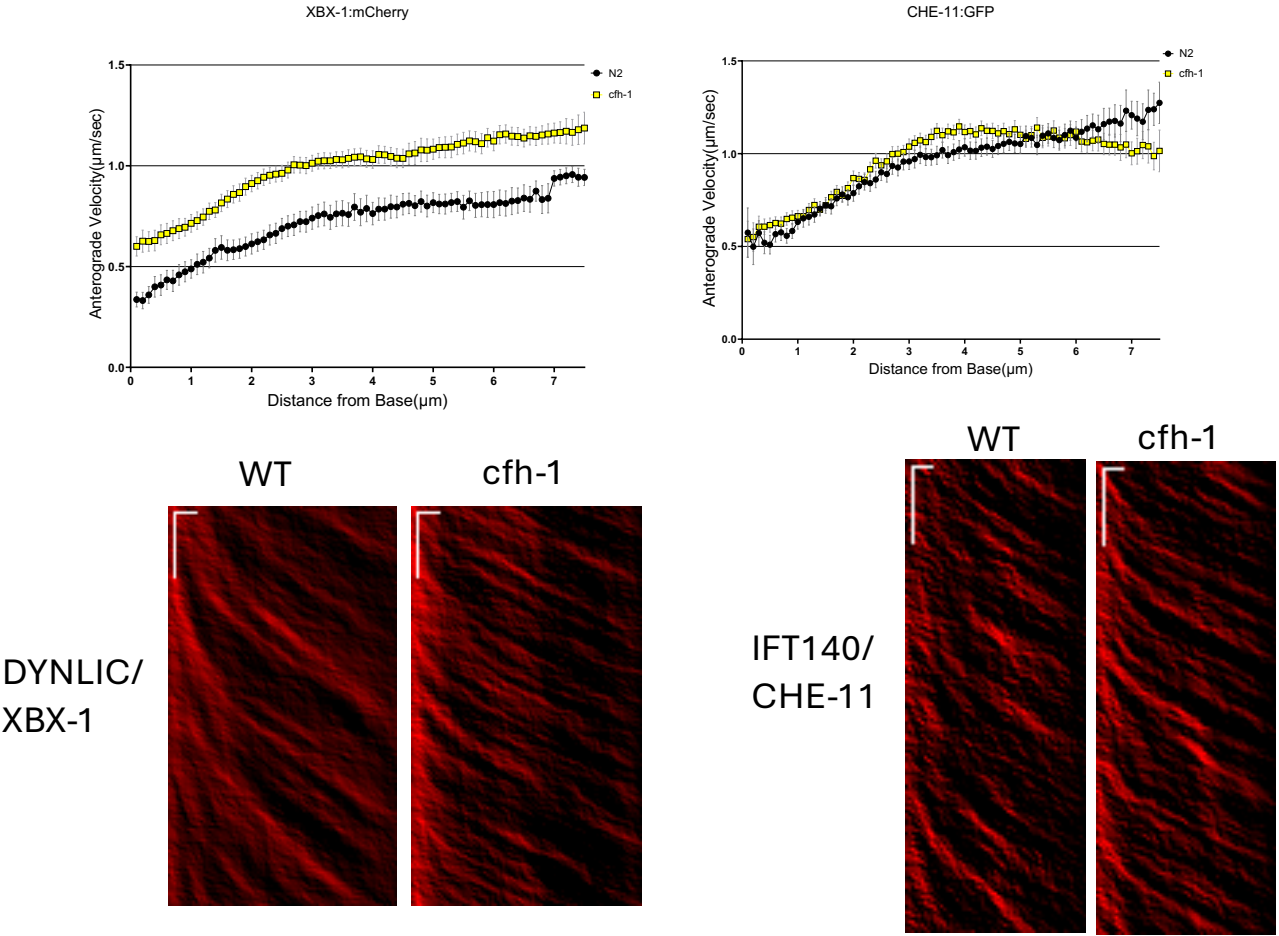

Figure S2

A

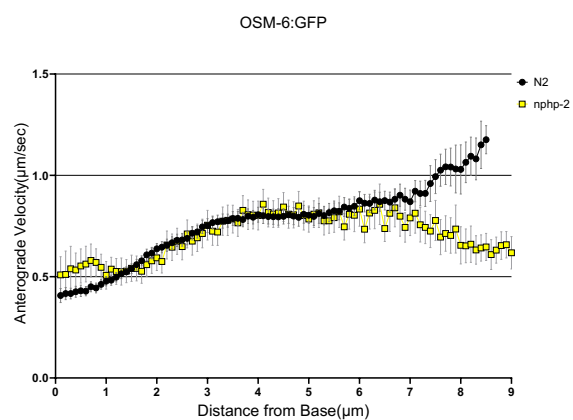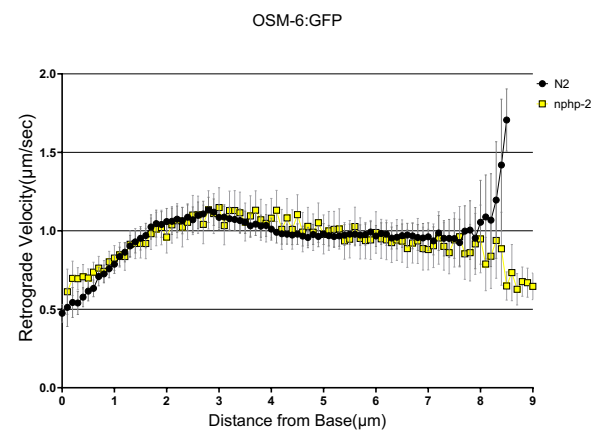

B

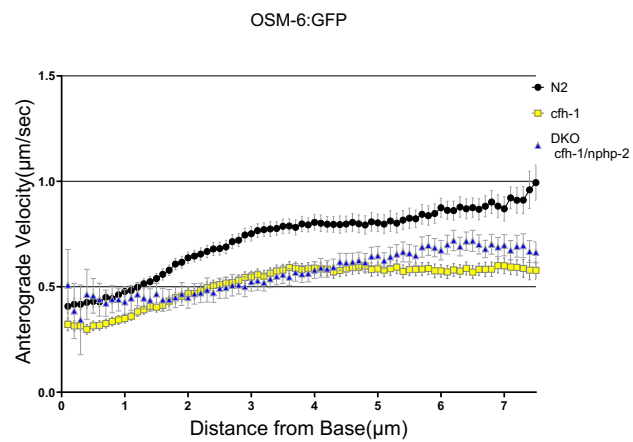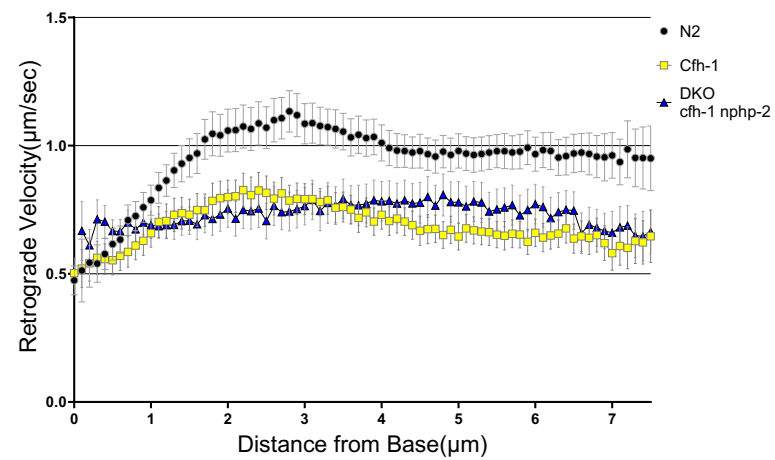

Figure S3

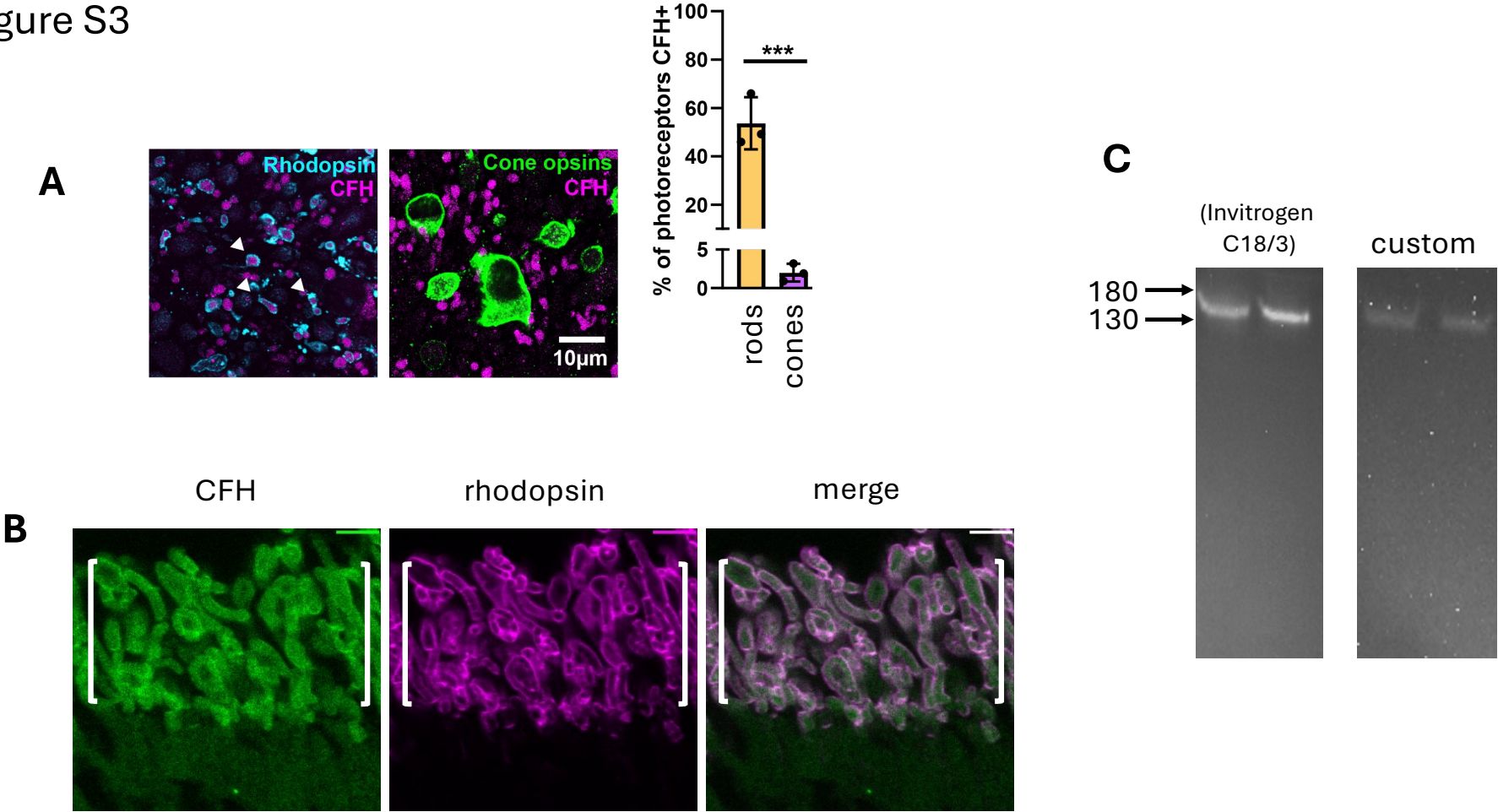

Supplement: Supplement 2 — Figure S1. Comparison of anterograde IFT of dynein light-intermediate chain XBX-1 and IFT-A component IFT140/CHE-11 in WT and cfh-1 mutant C. elegans day 4 adults. A, Plots of anterograde IFT velocity vs. distance from cilia base for dynein light-intermediate chain XBX-1 (left) and IFT-A component IFT140/CHE-11 in phasmid sensory neurons of WT(black) and cfh-1 mutant (yellow) animals. Note that IFT velocities for IFT140/CHE-11 are similar in WT and cfh-1(em14) animals while XBX-1 IFT is faster in cfh-1 mutant animals than in WT. B, Representative kymograph plots upon which the data are based. Horizonal scale bars represent 1μm and vertical scale bars indicate a 5 second interval. Figure S2. NPHP-2 does not affect IFT rates. A, Comparison of anterograde and retrograde IFT of IFT52/OSM-6 in WT and nphp-2(gk653) mutant C. elegans day 1 adults. Note that there is little or no difference in IFT52/OSM-6::GFP IFT in WT and nphp-2 mutant animals. B, Comparison of anterograde and retrograde IFT of IFT52/OSM-6 in WT, cfh-1(em14) and nphp-2 (gk653); cfh-1(em14) double mutant day 4 adults. Note that there is little or no difference in IFT52/OSM-6::GFP IFT in cfh-1 and cfh-1;nphp-2 mutant animals. Figure S3. CFH expression associated with human rod and cone photoreceptors A, Immunofluorescence with antibodies to CFH protein (magenta) and rhodopsin (cyan) in left panel or SWopsin and LWMWopsin (green, right panel) in 190 day old human retinal organoids. White arrows point to examples of rods with CFH. Right, scatter plot showing that a significantly higher proportion of rods contain CFH compared to cones in human organoids. Asterisks indicate significant differences detected by an ANOVA followed by a Tukey HSD test, where *** = p < 0.001. Error bars represent standard deviation of the mean. B, Antibody staining of human retina sections reveals CFH staining of photoreceptor outer segments. Also show is rhodopsin staining to indicate rod outer segments (brackets). Scale bar is 5μm. [file media-2.pdf]
